# Strategies for overcoming bottlenecks in allogeneic CAR-T cell therapy

**DOI:** 10.3389/fimmu.2023.1199145

**Published:** 2023-07-24

**Authors:** Zixin Lv, Feifei Luo, Yiwei Chu

**Affiliations:** ^1^ Department of Immunology, School of Basic Medical Sciences, and Institutes of Biomedical Sciences, Fudan University, Shanghai, China; ^2^ Biotherapy Research Center, Fudan University, Shanghai, China; ^3^ Department of Digestive Diseases, Huashan Hospital, Fudan University, Shanghai, China

**Keywords:** allogeneic CAR-T cell, gene-editing technology, non-gene editing technology, T cell subsets, pluripotent stem cell

## Abstract

Patient-derived autologous chimeric antigen receptor (CAR)-T cell therapy is a revolutionary breakthrough in immunotherapy and has made impressive progress in both preclinical and clinical studies. However, autologous CAR-T cells still have notable drawbacks in clinical manufacture, such as long production time, variable cell potency and possible manufacturing failures. Allogeneic CAR-T cell therapy is significantly superior to autologous CAR-T cell therapy in these aspects. The use of allogeneic CAR-T cell therapy may provide simplified manufacturing process and allow the creation of ‘off-the-shelf’ products, facilitating the treatments of various types of tumors at less delivery time. Nevertheless, severe graft-versus-host disease (GvHD) or host-mediated allorejection may occur in the allogeneic setting, implying that addressing these two critical issues is urgent for the clinical application of allogeneic CAR-T cell therapy. In this review, we summarize the current approaches to overcome GvHD and host rejection, which empower allogeneic CAR-T cell therapy with a broader future.

## 1 Introduction

**Figure 1:**
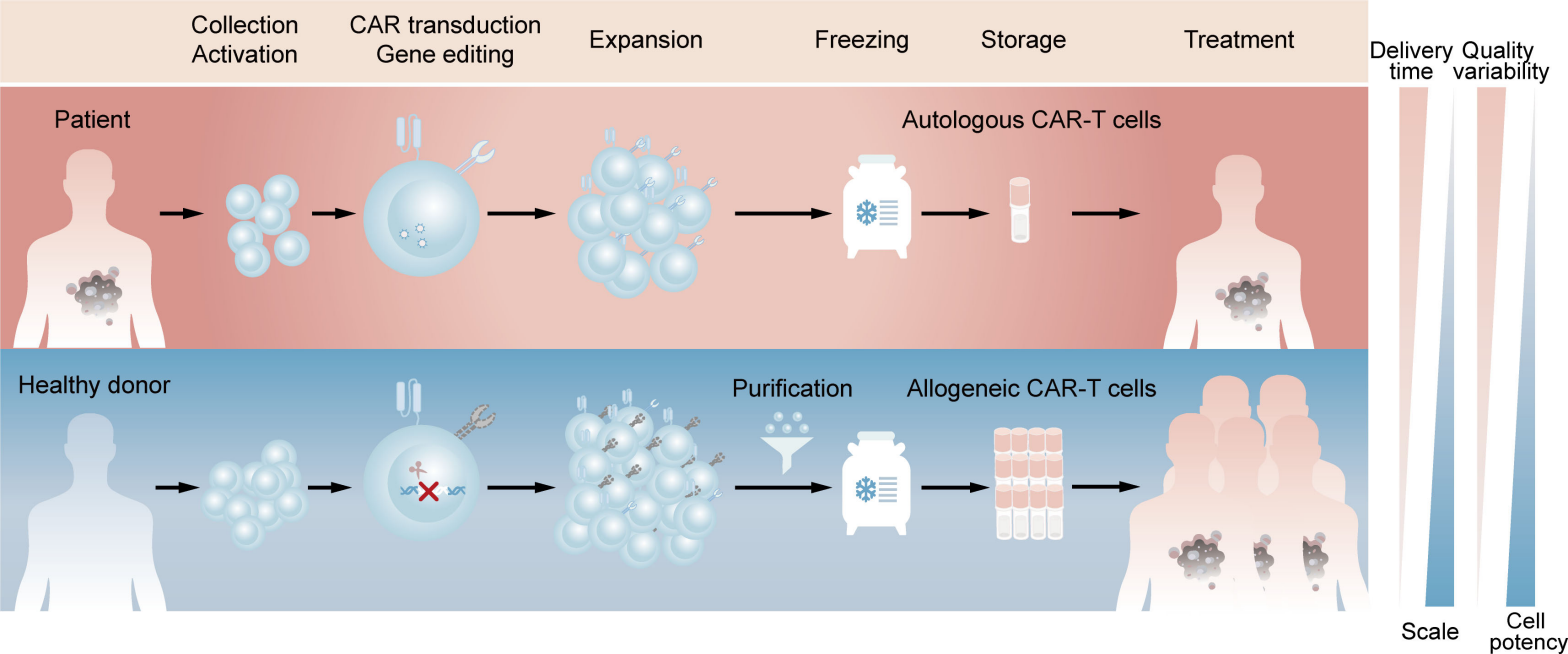
Comparison of clinic manufacturing process between autologous and allogeneic CAR-T cells. The above (red) represents the process flows of patient-derived autologous CAR-T cells. T lymphocytes are firstly enriched and activated from peripheral blood mononuclear cells. Afterwards, CARs are introduced by viral transduction and the obtained CAR-T cells are expanded and frozen, which are available for the patient’s own use after reviving. The below (blue) represents the process flows of healthy donor-derived allogeneic CAR-T cells. T lymphocytes are same enriched and activated, followed by elimination of TCR at the time of CAR introduction. The TCR-negative CAR-T cells are then purified from expanded cell population and the final frozen cells are available for different patients.

Chimeric antigen receptor (CAR)-T cell therapy is an innovative approach in cancer treatment, which has achieved great success in hematological malignancies and emerged as a promising treatment modality against solid tumors (1, 2). With the sixth CAR-T product approved by FDA and likely more to come (3), autologous CAR-T cell therapy, in which a patient’s own T cells are genetically engineered *in vitro* and then infused back into the patient, has demonstrated its importance in clinical applications. However, autologous CAR-T products inevitably raise challenging issues due to the customized and complex process flows. Since the clinical manufacture of CAR-T cells takes 1 to 3 weeks from collecting the patient’s blood to the final harvest of qualified cell products (4), patients with rapidly progressive tumors may lose access to CAR-T administration. Besides, the personalized pattern of autologous products means that CAR-T products may derive from different batches even for the same patient, which represents complicated processes and excessive costs (5). Moreover, T cell dysfunction caused by the healthy level and previous treatment of patients, as well as flawed processes in clinic manufacture, can contribute to the possibility of manufacturing failure (6, 7). Allogeneic CAR-T cells, which are also known as ‘off-the-shelf’ CAR-T cells, is an alternative to potentially overcome all these issues. For example, allogeneic CAR-T cells with starting materials sourced from healthy donors have the potential to increase predictable efficacy and treat all eligible patients. Compared with autologous CAR-T cells, a large number of allogeneic CAR-T cells can be produced in a single manufacturing run, meeting the needs of treatment for multiple patients and the requirements for cost reduction due to scale-up production. In addition, elimination the delay of treatment through reviving pre-frozen allogeneic cells in time whenever patients need may improve the clinical outcomes (**Figure 1**) (8, 9).

Although allogeneic CAR-T cell therapy has such advantages, it still remains hurdles to hamper its practical application. From an immunological perspective, donor CAR-T cells may attack host tissues to induce graft-versus-host disease (GvHD), and may be recognized by host immune system to cause host-mediated allorejection (8, 10). In this review, we describe the present strategies to address these risks through technology approaches in αβ T cells or using different cell types. We also discuss clinical transformation of universal allogeneic CAR-T cells based on the current strategies.

## 2 Modifying αβ T cells

Human leukocyte antigen (HLA) mismatch between donor and recipient is the major cause of GvHD and host immune rejection. The HLA system is a cluster of gene complex located on chromosome 6 in humans, which encodes cell-surface proteins responsible for presenting peptides to the αβ T-cell receptor (TCR) on T cells (11). HLA is known to be the most polymorphic gene region mainly caused by multiple alleles and codominance, and thus leads to high possibility of HLA mismatch in populations. The αβ TCR from donor CAR-T cells, which is composed of α- and β-chains, is able to recognize foreign HLA molecules with peptides restrictively, hence inducing acute or chronic GvHD. In turn, host αβ T cells may attack allogeneic CAR-T cells in an HLA-restricted way and trigger host rejection. Natural killer (NK) cells also participate in the rejection of donor CAR-T cells through tilting the dynamic balance between signals from cell surface activating and inhibitory receptors. Upon the unique recognition of inhibitory receptors and their ligands HLA class I (HLA-I) molecules, NK cells acquire immune tolerance to self, which means that mismatch between donor HLA-I and inhibitory receptors of recipient NK cells will weaken the transmitted inhibitory signal and then activate NK cells (12). In addition to cellular rejection, antibody-mediated rejection driven by donor-specific antibodies (DSAs) is also associated with graft damage after transplantation. DSAs contain antibodies against recipient’s HLA antigens and non-HLA antigens like minor histocompatibility antigens and autoantigens. In clinical trials of allogeneic cell therapy, DSAs were reported to induce cell killing through antibody-mediated cellular cytotoxicity (ADCC) and complement-dependent cytotoxicity (CDC) (13). Therefore, efforts are necessarily needed to solve these two problems in allogeneic CAR-T cell therapy.

### 2.1 Using gene editing technology

Modification of allogeneic CAR-T cells via gene editing technology is one of the major strategies to avoid GvHD and host-mediated allorejection. Gene editing technology is based on two mechanisms: double-strand breaks can be repaired to mediate gene knock-in through homologous recombination or gene knockout through non-homologous end joining when template strand is introduced or not, respectively (14). Currently, a series of powerful gene editing tools, such as zinc-finger nucleases (ZFNs), transcription activator-like effector nucleases (TALENs), and clustered regularly interspaced short palindromic repeats-CRISPR-associated protein 9 (CRISPR-Cas9), have played a role in preclinical and clinical studies of allogeneic CAR-T cells (15–17).

**Figure 2:**
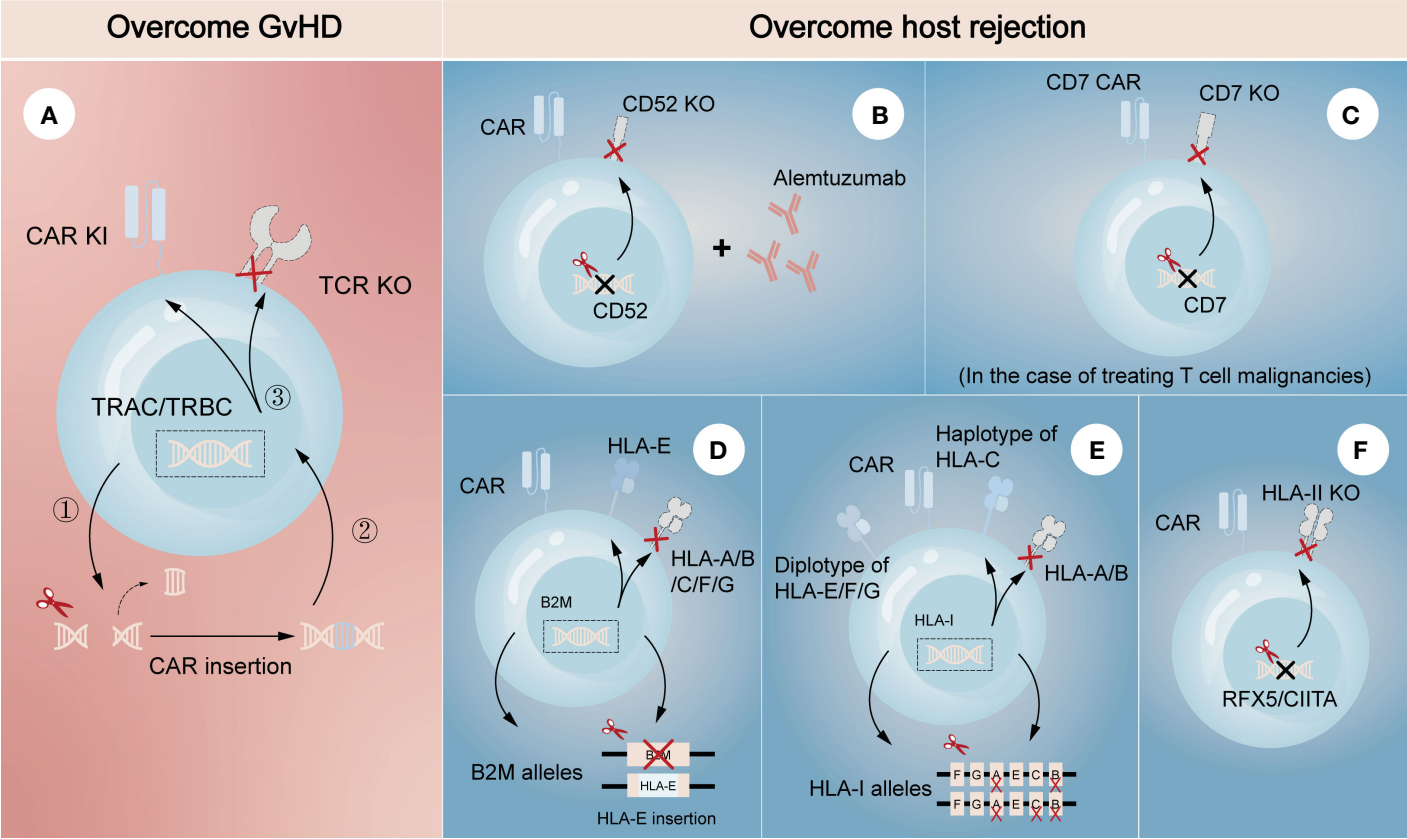
Gene editing technologies for overcoming GvHD and host-mediated allorejection. The left **(A)** represents the strategy to overcome GvHD that inserting CAR gene into the TRAC/TRBC loci. The right **(B–F)** represents the strategies to overcome host rejection, showing a combination strategy by removing CD52 in CAR-T cells with lymphodepletion through Alemtuzumab **(B)**, knockout of CD7 in CAR-T cells when treating CD7-positive T cell malignancies **(C)**, HLA-E insertion in one allele of B2M while another allele is knocked out **(D)**, construction of HLA-C-retained cells that disrupts both HLA-A and HLA-B alleles **(E)**, and knockout of transcription factors like RFX5/CIITA to achieve elimination of HLA-II **(F)**. TRAC, TCR α constant region; TRBC, TCR β constant region.

Genetically editing donor CAR-T cells to eliminate endogenous αβ TCR may effectively prevent TCR-mediated alloreactivity without compromising CAR-dependent function. Torikai et al. (15) first proposed that TCR-negative allogeneic anti-CD19 CAR-T cells, in which αβ TCR was deleted through ZFN-driven gene editing, showed the expected cytotoxicity of CD19 redirection without responding to TCR stimulation. This pioneering work introduced gene editing technology into allogeneic CAR-T cells, paving the way for its further use by utilizing different editing tools. More recently, a two-in-one strategy that inserting CAR gene into TCR α constant region (TRAC) through CRISPR system was designed (**Figure 2A**) (18). The produced allogeneic CAR-T cells lacked the unwanted graft-versus-host response, and were protected from the side effects of CAR transduction, such as virus usage and random integration. This two-in-one strategy further suggested that integrating CAR gene into targeted gene locus through gene editing may be a promising approach to both avoid the defects of CAR transduction and achieve the desired goal of targeted gene knockout. For example, CAR insertion in PDCD1 locus may resist exhaustion of CAR-T cells (19, 20).

Several strategies have also been developed to escape host rejection. One of the most commonly used in clinical settings is disrupting CD52 in donor CAR-T cells combined with lymphodepletion through Alemtuzumab, a humanized anti-CD52 monoclonal antibody (**Figure 2B**) (21, 22). CD52 is widely distributed on the surface of lymphocytes and many other hematopoietic cells, so pretreatment with Alemtuzumab can prevent host rejection and facilitate the expansion and survival of infused CD52-decifient CAR-T cells, which are resistant to Alemtuzumab. Since HLA-I molecules are expressed on the surface of most nucleated cells, abrogating their expression in CAR-T cells is another feasible strategy to evade the recognition by the recipient T cells. Disruption of β2-microglobulin (B2M) gene was reported to efficiently eliminate the expression of HLA-I molecules, as B2M is a highly-conserved component of HLA-I heterodimer. Nevertheless, the removal of HLA-I may lead to the activation and killing of NK cells, suggesting the limitations of direct deletion. Therefore, different modification methods were proposed to guarantee no ‘missing-self’ response by host NK cells. One is to express a single-chain HLA-E with minimal polymorphism fused to the B2M locus, which can effectively prevents killing of NKG2A^+^ NK cells (**Figure 2D**) (23, 24). Another is to construct an inventory containing HLA-C-retained cells that disrupts both HLA-A and HLA-B alleles. An induced pluripotent stem cell bank containing 12 lines of HLA-C-retained cells was found to be immunocompatible with >90% of the world population (25), demonstrating the construction of a cell inventory is potentially viable (**Figure 2E**). While not expressed in naive T cells, HLA class II (HLA-II) molecules are upregulated in activated T cells under the control of several trans-acting regulatory factors such as RFX5 and CIITA (26–28). Previous studies found that mutations in these regulatory genes led to lack of HLA-II antigen expression and CD4^+^ T cell-dependent immune response (29), indicating depletion of them would be a strategy to overcome host rejection caused by HLA-II-restricted recognition (**Figure 2F**). In the case of treating T cell malignancies, CD7-targeting allogeneic CAR-T cells with genetic modifications of CD7 depletion are also an encouraging approach to resist fratricide in addition to T cell-mediated rejection (**Figure 2C**) (30). As CD5 is also a pan-T marker acting as an inhibitory regulator of TCR signaling and regularly expresses on ~85% of T cell malignancies (31), it can also be an attractive target against T cell malignancies in allogeneic CAR-T therapy.

Combination of these strategies to overcome both host-mediated allorejection and GvHD has been intensively studied in the field of allogeneic CAR-T therapy (16, 19, 32). For instance, a recent study successfully generated CAR-T cells with immune-evasive properties by inserting CAR and HLA-E gene into TCR and B2M loci, respectively (33). These engineered cells were proved to evade the attack of T cells and NK cells, as well as extend their antitumor activity and persistence both *in vitro* and *in vivo*. The success of combination strategy in preclinical models enables the use of allogeneic T cells for adoptive T cell therapy, supporting the translation of research into clinical practice. To date, a number of clinical trials using gene-edited allogeneic T cells as the source of effector cells for CAR-T cell therapy are underway worldwide, most of which target hematological malignancies (34–36). UCART19 is the first-in-class allogeneic CAR-T product developed for the treatment of CD19-positive hematological malignancies, which is engineered to eliminate the expression of TCR and CD52 through TALEN system. A clinical trial (NCT02746952) based on UCART19 showed great expansion and antitumor activity of CAR-T cells in patients with relapsed or refractory B-cell acute lymphoblastic leukemia, in which only 2 patients developed grade 1 acute cutaneous GvHD (37). Many other clinical trials have also shown no or minimal GvHD and host rejection in gene-edited allogeneic CAR-T products, indicating the safety in clinical applications.

### 2.2 Using non-gene editing technology

Although gene editing technology is an excellent tool to modify allogeneic T cells, it actually carries various potential risks associated with carcinogenicity and chromosomal abnormalities. CRISPR-Cas9 editing was reported to potentially trigger p53-mediated programmed cell death, while downregulation of p53 made the cells more likely to be edited (38, 39). The findings suggest that p53-mutant cells are easier to be edited, therefore raising the risk of cell carcinogenesis. Moreover, cells with mutations in other cancer driver genes, such as the KRAS gene, have a competitive advantage after CRISPR-Cas9 editing (40). Numerical or structural chromosomal abnormalities are also hidden dangers of CRISPR-Cas9 editing (41, 42). CRISPR-Cas9 cleavage may cause chromosomal aberrations in human T cells, including chromosomal truncations, translocations and frequent aneuploidy (43, 44). All the reports point that global detection is needed to eliminate potential risks if CRISPR-Cas9 gene-edited cells are used for treatment. As an alternative, non-gene editing technologies have been explored to mitigate the risk of GvHD and host immune rejection.

**Figure 3:**
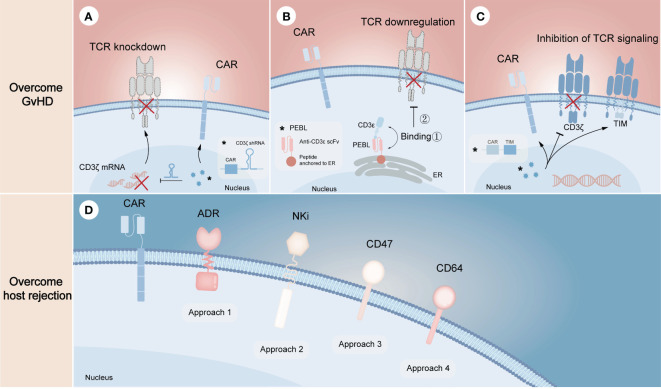
Non-gene editing technologies for overcoming GvHD and host-mediated allorejection. The above **(A–C)** represents the strategies to overcome GvHD, illustrating the knockdown of TCR complex through CD3ζ mRNA silencing **(A)**, the downregulation of TCR complex through expressing a CD3ϵ-specific PEBL that retaining the complex in cytoplasm **(B)**, and the inhibition of TCR signaling by expressing TIM molecules that can competitively replace CD3ζ when binding αβ TCR **(C)**. The below **(D)** represents the strategies to overcome host rejection, in which approach 1-4 show the expression of ADR for evading the killing of host T and NK cells, the expression of NKi to inhibit the activation of host NK cells and thus to reduce NK cell-mediated cytotoxicity, the overexpression of CD47 to escape host NK cell-mediated rejection, the overexpression of CD64 to escape antibody-mediated rejection, respectively. The symbol “*” represents the general schematic of a molecule (e.g., labeled virus particles (blue) in **A**) and its specific structure or composition (e.g., labeled white box in **A**).

Unlike gene editing technology that performs precise genetic manipulation in TCR locus to avoid alloreactivity of donor CAR-T cells, non-gene editing technology prefers to disrupt the downstream mechanisms of action. Since the formation of the TCR/CD3 complex is required for antigen recognition and downstream signal generation (45), inhibition of its assembly or cell surface expression may effectively prevent endogenous TCR-driven T cell activation. From the mRNA level, knocking down any subunit of TCR/CD3 complex through specific short hairpin RNA (shRNA) is one of the effective strategies to inhibit this complex formation (**Figure 3A**). On this basis, CYAD-211, a non-gene edited allogeneic CAR-T product that co-expresses CD3ζ shRNA with BCMA CAR via a single viral vector, was subsequently explored. A phase 1 trial (NCT04613557) evaluating CYAD-211 in adult patients with relapsed or refractory multiple myeloma showed an encouraging antitumor effect with a favorable safety profile (46), suggesting that knockdown of components in the complex is feasible. Several means of affecting the formation of TCR/CD3 complex on cell surface at the protein level have also been proposed. As the complex is assembled in the endoplasmic reticulum (ER), a specific protein expression blocker (PEBL) that consists of anti-CD3ϵ scFv and peptide predicted to anchor to ER was designed, thereby retaining the complex intracellularly (**Figure 3B**) (47, 48). Besides, expression of a TCR inhibitory molecule (TIM) that consists of a truncated CD3ζ peptide can result in dominant suppressive effects in TCR downstream signal through competitively form TCR/TIM complex (**Figure 3C**). By co-expressing a NKG2D CAR and a TIM, the CAR-T product CYAD-101 was proved to have antitumor activity without GvHD in mouse models and clinical trials (NCT03692429) (49, 50).

Resistance of allogeneic CAR-T cells to the recipient’s immune system requires eliminating alloreactive response mediated by T cells, NK cells and DSAs (**Figure 3D**). Activated lymphocytes temporarily upregulate the expression of several surface markers to provide additional costimulatory signals (51, 52), which can be targeted to eliminate host T cells and NK cells activated by allogeneic T cells, thereby preventing allogeneic T cells from being rejected. Based on the proof of principles, an alloimmune defense receptor (ADR) that consisted of an extracellular 4-1BB ligand-derived recognizing fragment fused to an intracellular CD3ζ chain was proposed to apply to allogeneic CAR-T cell therapy (53). The expression of ADR was shown to effectively resist host rejection and fratricide as the expression of ADR could downregulate or mask their own 4-1BB. As NK cells can be activated by ‘missing self’ recognition, Hu et al. (30) generated an NK cell inhibitory receptor (NKi), which was composed of the EC1-EC2 extracellular domain of E-cadherin and the CD28 intracellular domain. Due to the fact that E-cadherin negatively regulates NK cell function through KLRG1 binding and that CD28 intracellular domain enhances the inhibitory effect of NKi (54), the incorporation of NKi into CAR-T cells resulted in a reduction in NK cell-mediated cytotoxicity. Besides, previous studies showed that upregulation of CD47 expression specifically prevented NK cells from killing allogeneic stem cells and cells with HLA-I deletion, illustrating that ectopic expression of CD47 in CAR-T cells may be a novel strategy to solve NK cell-mediated rejection (55, 56). However, it still needs to be further evaluated in preclinical models. More recently, overexpression of CD64 was found to protect CAR-T cells from HLA and non-HLA antibody killing (13). Since CD64 is a high-affinity receptor for the fragment crystallizable (F_c_) region of IgG, CAR-T cells that overexpress CD64 can capture IgG F_c_ and make DSAs inaccessible to effector cells and complement, thus allowing CAR-T cells to evade ADCC and CDC.

## 3 Using different T cell subpopulations

Despite the fact that αβ T cells are the dominant source of effector cells in CAR-T cell therapy, their use in allogeneic settings still needs significant efforts. In addition to the manufacturing process required for traditional autologous CAR-T cells, allogeneic CAR-αβ T cells, whether modified via gene-editing or non-gene editing technologies, requires additional process to achieve the clearance of GvHD and host-mediated allorejection, as well as to ensure the clinical safety of the modified cells (9). Therefore, a number of studies attempt to broaden the source of effector cells to achieve a final product with less process and higher safety. Here we describe the advantages of novel T cell subsets available for CAR-T therapy over traditional αβ T cells (**Table 1**), and summarize the clinical progress of CAR-T therapy based on them (**Table 2**).

**Table 1:** Summary of allogeneic CAR-T products from different T cell sources.

CAR-T product	Target antigen	T cell source	Conditions	Phase	Reference
KUR-502	CD19	iNKT	Relapsed or refractory B-cell malignancies	Phase 1 (NCT00840853)	(57)
CD123CAR-DOTs	CD123	γδT	acute myeloid leukemia	Preclinical	(58)
ADI-002	GPC3	γδT	Hepatocellular carcinoma	Preclinical	(59)
ADI-001	CD20	γδT	B cell malignancies	Phase 1 (NCT04735471)	(60)
CTM-N2D	NKG2DL	γδT	Advanced solid tumors or hematological malignancies	Phase1 (NCT05302037)	
CD19-CAR-DNT	CD19	DNT	B-cell non-Hodgkin’s lymphoma	Phase1 (NCT05453669)	(61)
FT819	CD19	iPSC-derived TCRαβ-deficient T	Relapsed or refractory B-cell malignancies	Phase1 (NCT04629729)	(62)
CNTY-102	CD19 and CD22	iPSC-derived γδT	Relapsed or refractory B-cell malignancies	Preclinical	

**Table 2:** Comparation among different T cell sources.

T cell subset	Frequency in human peripheral blood T cells	MHC restriction	Advantages	Disadvantages
αβT	90%-95%	YES	Highly abundance; well-established system for clinical application	GvHD and host rejection; the need for multiple manipulations
iNKT	0.01%-1%	NO	Double killing effects; lack of GvHD; better tumor localization	Low frequency; host rejection
γδT	5%-10%	NO	Multiple mechanisms of action; lack of GvHD; better migration and infiltration; antigen cross-presentation	Low frequency; host rejection
DNT	1%-3%	NO	Multiple targeting; lack of GvHD and host rejection	Low frequency
MAIT	1%-10%	NO	Multiple mechanisms of action; lack of GvHD; effector memory-like phenotype	Low frequency; host rejection; immature expansion system

### 3.1 CAR-invariant natural killer T cells

Invariant NKT (iNKT) cells are a CD1d-restricted, TCR semi-invariant T cell subpopulation with TCR containing an invariant Vα24-Jα18 chain. The monomorphism of CD1d gives iNKT cells the ability to overcome HLA-restricted GvHD in the allogeneic settings without inactivating their endogenous TCR, thus providing a safer source of effector cells for the ‘off-the-shelf’ CAR-T platform (63). Preclinical models showed that CAR-NKT cells had better localization ability to tumor tissues than conventional CAR-T cells and played a dual killing effect by targeting CAR-positive tumor cells and CD1d-positive M2 or tumor cells to reduce the immune escape of tumor cells (64–66). Moreover, they were able to facilitate maturation of DC through CD40L/CD40 and TCR/CD1d signaling and thus indirectly activate CD8^+^ T cells (67). The first clinical trial based on autologous CAR-NKT cells was conducted previously (NCT03294954) (68). NKT cells were engineered to co-express a CAR that could specifically recognize GD2-expressing neuroblastoma and IL-15 that could support the survival of NKT cells. The GD2.CAR.15 NKT cells showed enhanced persistence, better tumor localization and improved tumor control without significant toxicity, which suggests the great potential of CAR-NKT cells over conventional CAR-T cells. In addition, donor-derived iNKT cells were proved to exert antitumor function without exacerbating GvHD after allogeneic hematopoietic cell transplantation, indicating the possibility for ‘off-the-shelf’ production and use (69).

Because of the relative rarity that iNKT cells account for only 0.01%-1% of peripheral blood T cells (70), expansion of them from donor peripheral blood mononuclear cells (PBMC) to therapeutic level is crucial for clinical use. Rotolo et al. (66) built a system in which TCRVα24Jα18^+^ lymphocytes were activated by CD3/CD28 beads and cultured with an aliquot of irradiated autologous mononuclear cells at a 1:1 ratio in the presence of IL-15, resulting in an average 750-fold expansion of 3rd CAR19-iNKT cells after 3 weeks. Ngai et al. (71) also found that co-culture of iNKT cells with an aliquot of αGalCer-pulsed irradiated autologous PBMC in the presence of IL2 and IL-21 led to an average 600-fold expansion and increased CD62L^+^ frequency in 10-12 days. Currently, a clinical study of allogeneic CD19 CAR-NKT cells for relapsed or refractory B-cell malignancies is underway (NCT00840853) (57). The NKT cells were modified to express a CD19-targeting CAR, IL-15, and shRNA targeting B2M and CD74 to downregulate HLA-I and HLA-II molecules, respectively. The initial results indicated that these allogeneic cells were well-tolerated and could mediate antitumor responses in patients.

### 3.2 CAR-γδ T cells

γδ T cells, whose TCR consists of γ- and δ-chain, are a subpopulation of T cells appearing in peripheral blood and barriers like intestine (72). Different from αβ T cells that recognize the MHC-peptide complex, γδ T cells play an important role in both innate and adaptive immune responses through multiple receptor-ligand interactions. In antitumor immunity, γδ T cells function in an MHC-independent manner of antigen recognition and tumor killing, as well as play an indirect role by activating B cells, αβ T cells and NK cells (73, 74). Multiple preclinical trials have found that the presence of γδ T cells after allogeneic stem cell transplantation do not induce GvHD (75, 76), a feature that makes them an attractive pool for allogeneic CAR-T therapy. A previous γδ T-based GD2 CAR-T study showed that CAR-γδ T cells could better migrate and infiltrate into tumor site, exert antitumor effects and cross-present antigens to tumor-infiltrating αβ T cells through uptake of released tumor-associated antigens (77). Other studies of CAR-γδ T cells also described several established production process of high-purity CAR-γδ T from PBMC (58, 59, 78). These harvested CAR-γδ T cells were shown to kill antigen-negative tumor cells without evidence of GvHD, indicating the ability to eliminate tumors even after antigen loss. All these characteristics mark their potential advantages over conventional CAR-αβ T cells. Currently, several allogeneic CAR-T cell therapies based on γδ T cells (NCT04735471, NCT05302037) are in phase 1 clinical trials for the treatment of hematological malignancies or solid tumors (60).

### 3.3 CAR-double negative T cells

Double negative T (DNT) cells are a small subset of mature T cells that comprise approximately 1%-3% of T lymphocytes in human peripheral blood (79). Distinguished from conventional T cells and NKT cells, DNT cells lack both CD4 and CD8 in addition to not binding CD1d tetramers. DNT cells can recognize tumor cells through NKG2D or DNAM-1 and exert antitumor functions through cytokines or Fas/FasL signaling pathway (80). This MHC-independent and multi-targeted mechanism of action allows DNT cells to not cause GvHD and to kill tumor cells that do not express MHC molecules. Various preclinical models demonstrate that allogeneic DNT cells can target a range of tumor types and mediate significant cytotoxicity without triggering GvHD and host rejection (81–84). For example, healthy donor-derived DNT cells significantly inhibited the growth of late-stage lung cancer xenografts in mouse models (85). A Phase 1 clinical trial (NCT03027102) using healthy donor-derived DNT cells also validated objective responses without observed toxicity to normal tissues in patients with acute myeloid leukemia (86). These studies all suggest that DNT cells, which do not induce alloreactivity without modification, have the properties to be used in allogeneic settings.

Although the frequency of DNT cells in peripheral blood is relatively low, large-scale expansion to therapeutic levels can be reached under GMP conditions. Briefly, CD4- and CD8-negative PBMC are sorted and cultured in the presence of IL-2 and CD3 mAbs for 17 days, which allows the harvested DNT cells to reach an average fold and purity of 1558 and 91.9%, respectively (82, 83). As IL-33 has recently been reported to promote the proliferation and survival of DNT cells *in vitro* (87), adding IL-33 may be an optimized strategy to improve the current clinical expansion system for DNT cells. However, an optimal dosage-frequency regimen for IL-33 requires further exploration. More recently, Vasic et al. (88) generated CAR19-DNT cells by transducing CD19-specific CAR into DNT cells. These modified cells efficiently inhibited tumor growth with no off-tumor toxicity in mouse models, while they were able to evade the alloreactivity of mouse-derived T cells. Therefore, DNT cells have met several requirements for clinical application of allogeneic CAR-T products, including the efficacy, lack of alloreactivity and large-scale production.

### 3.4 CAR-mucosal-associated invariant T cells

Mucosal-associated invariant T (MAIT) cells are an evolutionarily conserved, unconventional T cell subpopulation, of which approximately 90% are phenotypically CD161^+^CD8^+^. MAIT cells have the unique semi-constant TCR that consists of TRAV1 combined with three kinds of TRAJ (TRAJ33, TRAJ12, TRAJ20) and a limited repertoire of β chains in human (89). The TCR can recognize modified derivatives from vitamin B2 synthesis pathway presented by MHC class I-related molecule MR1 on APCs, promoting the proliferation and secretion of cytokines, cytotoxic molecules and chemokines of MAIT in the face of most microorganisms. Moreover, they express several NK activating receptors, which act as a second pathway to initiate response. In antitumor immunity, MAIT cells can kill tumor cells via TCR or NK activating receptors, meanwhile boosting DC through upregulating CD40L and inhibiting TAM and MDSC through binding MR1 and NK ligand (90). MAIT cells are distributed in peripheral blood, liver, lung and intestinal lamina propria (91). Previous studies have showed that CD161^+^CD8^+^ MAIT cells upregulate tissue-specific chemokine receptors like CXCL6, CCR6 and CCR9 (92), the reason that they are highly enriched in non-lymphoid organs through tissue homing. Besides, MAIT cells present an effector memory-like phenotype and can produce IFN-γ and granzyme B upon stimulation. Since MR1 is a conserved molecule, MAIT cells are devoid of alloreactive potential, as confirmed by their failure to proliferate in response to alloantigen stimulation *in vitro* and to expand or participate in tissue damage during GvHD *in vivo* (93). This potential has also been proved by clinical data showing that the number of engrafted allogeneic MAIT cells is positively correlated with improved survival and less allogeneic adverse events (94). All the intrinsic characteristics of MAIT cells, including multiple targeting, memory phenotype and lack of alloreactivity, make them candidates for allogeneic cell therapy against cancer.

As engineering immune cells with CAR is an effective measure to enhance antitumor function, Dogan et al. (95) constructed anti-CD19 and anti-HER2 CAR-MAIT cells to evaluate their efficacy while compared with conventional CAR-T cells. They found CAR-MAIT cells showed similar or even higher cytotoxicity but lower proinflammatory cytokine secretion compared with conventional CAR-T cells *in vitro* assays, indicating MAIT cells may represent safer and more effective effector cells for CAR-T cell therapy. However, the efficacy and safety evaluation of CAR-MAIT cells *in vivo* and in allogeneic settings have not been studied yet, which means more efforts are needed to explore the allogeneic CAR-MAIT therapy. In order to apply allogeneic CAR-MAIT cells in clinical conditions, a key issue is to achieve efficient expansion of MAIT cells. There are two expansion protocols for MAIT cells. The first is co-culture MAIT cells with artificial APCs generated by immobilizing antigen-loaded MR1 tetramers and CD28 mAbs on cell-size latex beads (96). The protocol leads to a 60-fold expansion of pure MAIT cells in 3 weeks. Another is co-culture MAIT cells with autologous PBMC at a ratio of 1:10 in a serum replacement-supplemented medium in the presence of IL-2, which results in a 200-fold expansion of high-purity MAIT cells in 3 weeks (97). For better clinical application, it is worthwhile to optimize the amplification system of MAIT cells.

## 4 Using pluripotent stem cells

Although the ‘off-the-shelf’ CAR-T cells have been a window of opportunity for the generalization and productization of CAR-T cell therapy, there are still inevitable drawbacks in process flows. Given the nature of T cells themselves, CAR-T cells undergo limited expansion and gradual transition to exhausted phenotypes in manufacturing (4), which creates the need for repeated collection of T cells from different donors as source materials and thus leads to heterogeneity of CAR-T products. In addition, CAR gene insertion or gene editing at the T cell level is technically challenging and requires complex safety assessments to prevent adverse events such as chromosomal abnormalities and oncogenic mutations (5).

Pluripotent stem cells (PSCs) such as induced pluripotent stem cells (iPSCs) and embryonic stem cells (ESCs) may be a better choice of source materials to solve the problems in clinic manufacture. In contrast to T cells that require large-scale quality control to avoid adverse events, the successfully engineered PSCs with security can be select easily to create a master cell bank for downstream use (98). Besides, gene editing and CAR transduction do not affect the properties of PSCs to proliferate indefinitely. A qualified master CAR-PSC cell bank can provide an inexhaustible source of starting cells, ensuring the generation of numerous homogeneous CAR-T cell products for a large number of patients (99). However, *in vitro* generation of mature T cells from PSCs that are phenotypically and functionally similar to classical TCRαβ^+^ T cells is a challenge to overcome.

**Figure 4:**
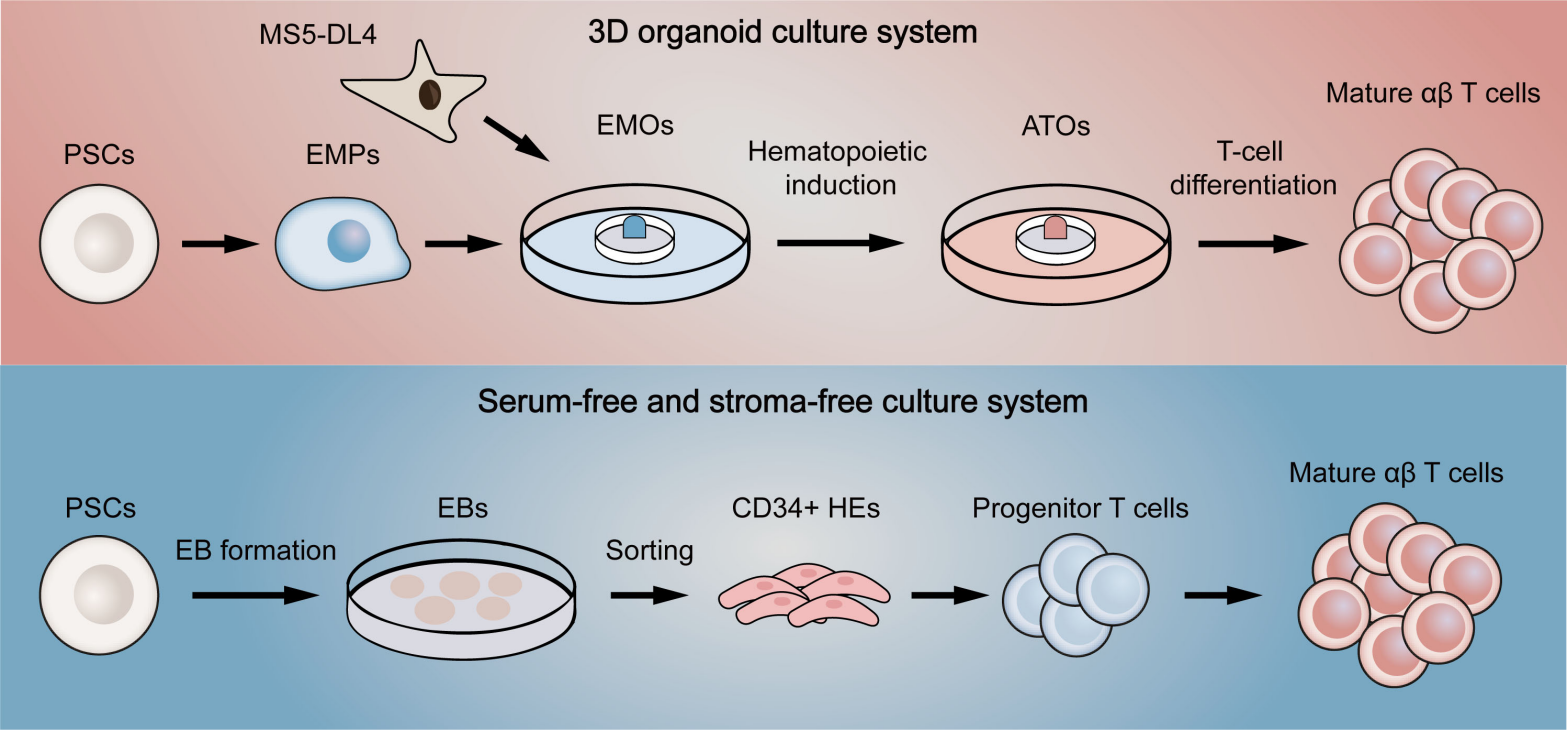
Two novel differentiation systems for generation T cells from pluripotent stem cells. The above (red) briefly summarizes the flow of the 3D organoid culture system. EMPs differentiated by PSCs are first co-cultured with DL4-expressing MS5 cells to form EMOs. After hematopoietic induction, ATOs are then formed. Finally, T-cell differentiation is fulfilled to generate single-positive T cells. The below (blue) represents the serum-free and stroma-free culture system. PSCs are first differentiated into EBs. CD34-positive HEs are then obtained by dissociating EBs, which later gradually differentiate into progenitor T cells, double-positive T cells and finally mature as single-positive T cells. EMPs, embryonic mesodermal progenitor cells; EMOs, embryonic mesodermal organoids; ATOs, artificial thymus organoids; EBs, embryoid bodies; HEs, hemogenic endothelial cells.

To address this problem, Schmitt et al. (100) first proposed a simple system of co-culture ESCs with OP9-DL1, a mouse bone marrow stromal cell line expressing Notch ligand delta-like 1 (DL1). Since it was previously reported that culture of ESCs on OP9 promoted the development of hematopoietic lineage and that DL1 signaling induced differentiation of hematopoietic stem cells into T cells (101, 102), ESCs were effectively differentiated into functional single-positive T cells in this system. Nevertheless, the hematopoietic progenitor cells obtained in the process were proved to have low engraftment levels and hematopoietic chimerism in transplantation (103, 104), reflecting that the conditions for hematopoietic differentiation still need refining. Therefore, several methods were used to improve conditions for definitive hematopoietic specification (105). For example, transcription factor respecification, optimized procedures for co-culture with stromal cells, and direct differentiation in a serum-free condition with cytokines or in a cytokine-free condition were all shown to facilitate definitive hematopoiesis (104, 106–108). Although these improved approaches partially yield mature T cells, there still remain a large number of T cells in precursor stage or with innate phenotype. Two novel methods were then designed to increase the quality of T cells in final harvests (**Figure 4**). One is to culture PSCs with MS5 cells expressing Delta-like-4 (DL4) in a 3D organoid culture system, a method that is readily deployable and technically advanced (109). The system successfully differentiated PSCs into high proportions of mature αβ T cells, which were transcriptionally similar to naive CD8^+^ T cells and produced cytokines in response to antigens. Besides, the system also permitted the generation of highly functional CAR-T cells from CAR-iPSCs (110), providing a platform for ‘off-the-shelf’ CAR-T therapy. Since co-culture with stromal cells is difficult in clinical manufacture, another method aims to generate T cells in a totally serum-free and stroma-free system that is clinically applicable and scalable. The approach replaced DL4-expressing stromal cells with DL4-Fc recombinant protein, eliminating the role of stromal cells in the culture system. More recently, some improvements to this system have also been reported. The first is to replace plate-bound DL4-Fc recombinant protein with mobile DL4-μbeads (111), providing a cell suspension culture applicable for scale up production. The second is coupling the system with histone methyltransferase EZH1 repression (112). The final harvested EZ-T cells showed similar molecular features to peripheral blood αβ T cells, and displayed effector and memory-like phenotypes after activation. Besides, CAR-T cells based on EZ-T exhibited robust antitumor activity in mouse models. In summary, mature CAR-T cells can be efficiently generated from PSCs nowadays, greatly facilitating the path to ‘off-the-shelf’ allogeneic CAR-T cell therapy.

## 5 Conclusion and perspectives

Although the elimination of GvHD and host-mediated rejection has enabled the clinical application of allogeneic CAR-T cell therapy, other challenges will also affect the safety and efficacy of the treatment. The clinical applications of allogeneic CAR-αβT products are made possible by a series of technologies inhibiting the occurrence of GvHD and host rejection, which prevent the damage to normal tissues and rapid clearance of allogeneic CAR-T cells in patients. However, neither gene knockout nor mRNA silencing can achieve complete removal of TCR from cell population (113). Additional depletion of remaining TCR^+^ cells is inevitable in clinic manufacture, which largely determines efficacy and toxicity. These complicated and time-consuming profiles limit the function of T cells, suggesting the importance to shorten *in vitro* manufacturing workflows by improving T cell culture technology or optimizing the processes. For other T cell subsets with low frequency and PSC-derived T cells, optimal strategies for expansion or differentiation systems are needed to explore. For example, further investigations of how gene networks manipulate key events in T cell development, such as T-cell lineage specification and commitment, β selection and positive selection, may help understand what shapes T cell fate. By promoting or inhibiting functions of some important genes in differentiation system, the number, phenotype and function in final products may be profoundly affected. In addition, previous research confirmed the importance of signals from TCR to maintain T cell homeostasis and long-term survival *in vivo* (114). A clinical study also showed limited efficiency and persistence of allogeneic CAR-T cells relative to autologous CAR-T cells (115). This deficiency not only affects the antitumor efficacy, but also implies the possibility of repeated dosing and consequent lymphodepletion, putting additional burdens on patients. Taken together, maintaining the long-term survival of allogeneic CAR-T cells *in vivo* will inevitably require the introduction of new modification strategies, such as modifying the CAR structure to attenuate CAR signaling or modulating the metabolic profiles to improve the persistence of allogeneic CAR-T cells.

Allogeneic CAR-T platform has facilitated the shift from customization to generalization of CAR-T cell therapy in cell source, meeting the urgent need of patients both in cell quality and delivery time. While many hurdles remain along the way, ‘off-the-shelf’ allogeneic CAR-T cell therapy has yielded promising results so far and has great possibilities waiting to be discovered due to their properties in addressing the pain points of autologous CAR-T therapy.

## Author contributions

ZL, FL, and YC wrote, critically reviewed, and edited the manuscript. All authors contributed to the article and approved the submitted version.
